# *Panax japonicus* C.A. Meyer: a comprehensive review on botany, phytochemistry, pharmacology, pharmacokinetics and authentication

**DOI:** 10.1186/s13020-023-00857-y

**Published:** 2023-11-10

**Authors:** Yuan Chen, Meiqi Liu, Jinli Wen, Zijie Yang, Guohui Li, Ying Cao, Lili Sun, Xiaoliang Ren

**Affiliations:** School of Chinese Materia Medica, Tianjin University of Traditional Chinese Medicine, Tianjin, 301617 China

**Keywords:** *Panax japonicus* C.A. Meyer, Phytochemistry, Pharmacology, Pharmacokinetic, Review, Zhujieshen

## Abstract

**Background:**

*Panax japonicu*s C.A. Meyer (Zhujieshen) is widely used in traditional medicine as a tonic hemostatic and anti-inflammatory agent in China, Japan, and Korea. Furthermore, it is used as an important substitute for ginseng roots by minority ethnic groups in China. The purpose of this review is to summarize the latest research on Zhujieshen in recent years, aiming at providing a systematic overview of the current knowledge, and perspectives for future research and exploitation.

**Main body:**

This review examines the research advances in botanical profile, phytochemicals, pharmacology, pharmacokinetics, and authentication of Zhujieshen. Various compounds have been reported as active components, mainly including saponins, volatile oils, and polysaccharides. Pharmacological investigations have demonstrated that Zhujieshen is an important herb with significant bioactivities, such as anti-inflammatory, hepato-protective, cardio-protective, neuro-protective, anti-tumor, anti-oxidant, anti-thrombotic and immunomodulatory activities.

**Conclusion:**

Currently, research on Zhujieshen is in the preliminary stages, and further research is required to understand the active compounds present and mechanisms of action. We hope that this comprehensive review of Zhujieshen will serve as a background for future research and exploitation.

## Introduction

*Panax japonicus* C.A. Meyer is a perennial herb belonging to the genus *Panax* in the Araliaceae family that mainly grows wild in China, Japan, and Korea [1–3]. *P. japonicus* is also known as “Zhujieshen” (竹节参 in Chinese) due to its bamboo-like long horizontally creeping rhizome. The rhizome of Zhujieshen used as a traditional Chinese medicine for a thousand years, is the king of herbs in traditional Tujia and Hmong medicine. It has been used as an important substitute for ginseng roots by minority ethnic groups [1, 4, 5]. Its pharmacological effects include promotion of blood flow, similarly to *P. notoginseng*, and as a strengthening tonic, similarly to *P. ginseng*. Zhujieshen has mainly been used as a tonic and hemostatic and anti-inflammatory agent in China for treatment of fracture, hematemesis, cough, bleeding wounds, arthralgia, and weakness after illness [3, 6]. The earliest history of Zhujieshen was recorded in *A Supplement to the Compendium of Materia Medica* (Qing Dynasty, *Ben Cao Gang Mu Shi Yi*, 本草纲目拾遗) in the Qing Dynasty. It has been recorded in the Pharmacopoeia of the People's Republic of China, which attributes various pharmacological activities to it.

Many recent scientific studies have reported on Zhujieshen, suggesting that it would be timely to conduct a systematic overview of the current knowledge. This review is intended to address research advances in botanical profile, phytochemicals, pharmacology, and pharmacokinetics of the extracts and isolated compounds from Zhujieshen. Moreover, this review aims to provide novel perspectives to further expand the therapeutic applications and exploitation of Zhujieshen for the treatment of various human diseases.

## Botany

**Fig.1:**
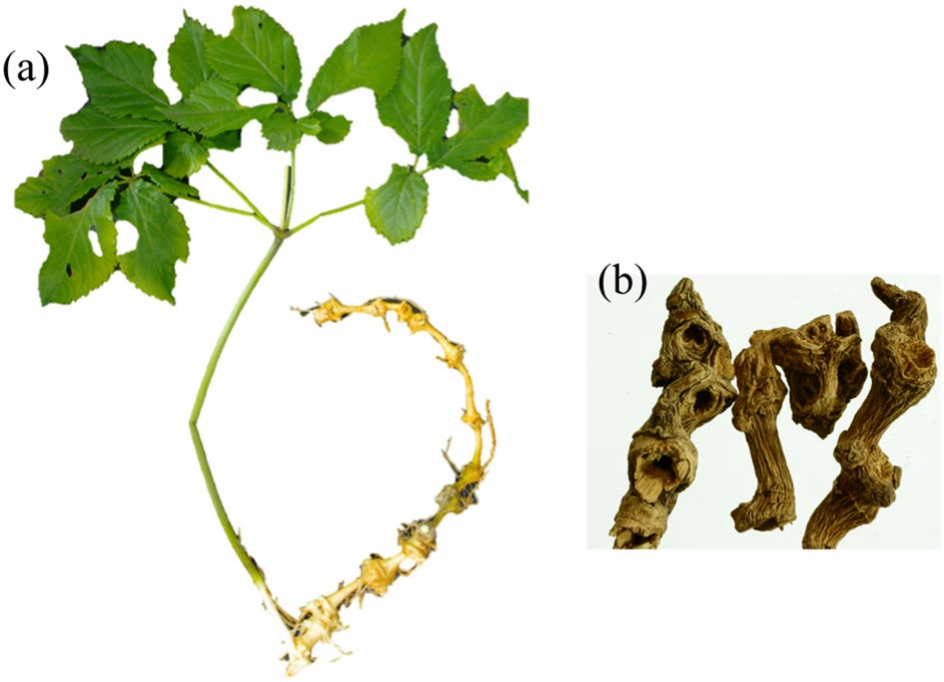
The medicinal plant Zhujieshen. **a** The whole plants, **b** dry bamboo-root-like rhizome

The bamboo-root-like rhizome of Zhujieshen (Fig. 1) is horizontal, plump, and white with short internodes, each node having a deep concave stem mark (concave eye). The stem is erect, 30–60 cm high, and cylindrical with longitudinal stripes. Three to five palmately compound leaves form one whorl at the top of the stem. The petiole is delicate and leaflets (usually 3 ~ 5) are obovate to obovate-elliptic, 8–12 cm long, and 3–5 cm wide. The apex of the leaflet is long and tapering. The base is wedge-shaped, extends downward and the edges are serrated. A few bristles are scattered on the upper vein, while the lower side is glabrous. The outermost pair of lateral leaflets is smaller. The microparticle is up to 2.5 cm long. An umbel is formed at the center of the top of the stem, about 15 cm in length, composed of many small flowers. The flowers are yellow-green, the calyx margin has five teeth, there are five petals, five stamens, and perigyny, and the ovary has two compartments. The fruits are kidney-shaped and bright red, each containing two seeds.

## Phytochemistry

**Fig.2:**
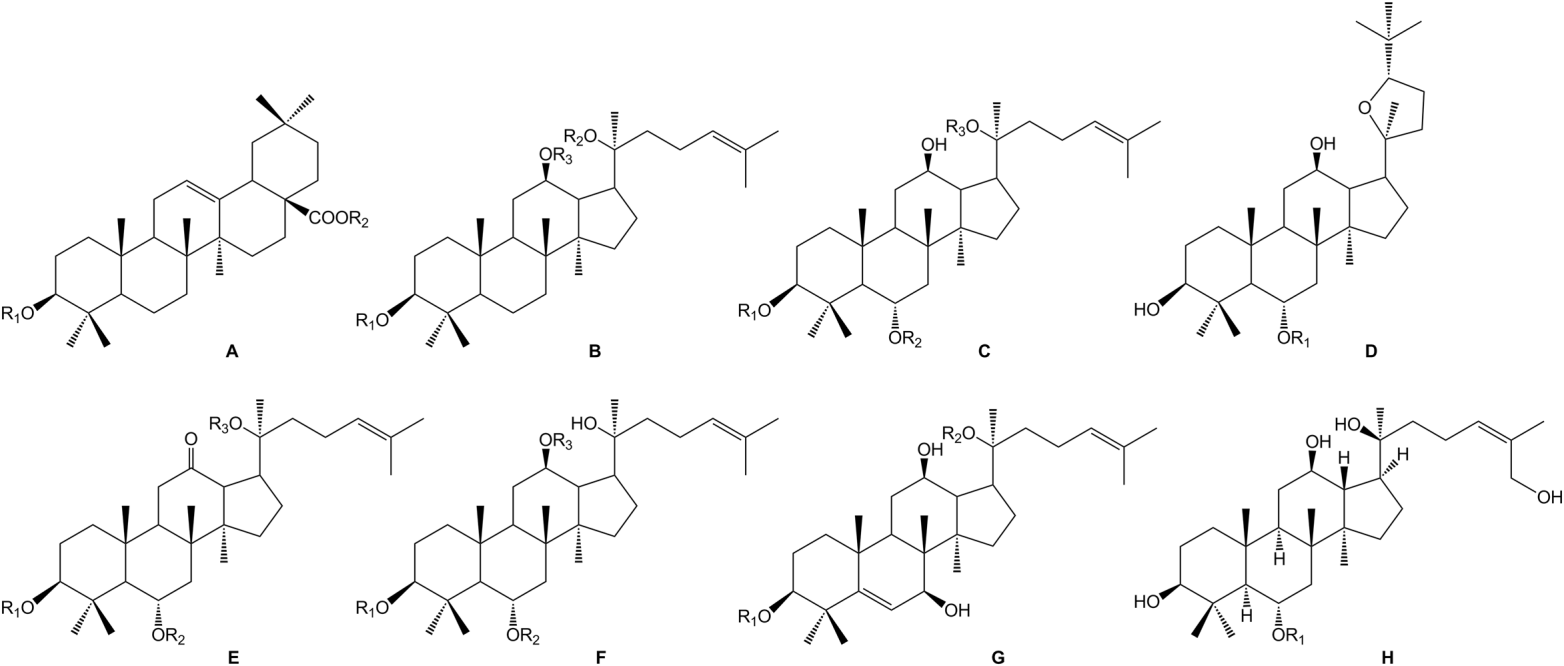
The parent nuclei of different saponins in Zhujieshen. **a** oleanolane type; **b** 20(S)-protopanaxadiol type; **c** 20(S)-protopanaxatriol type; **d** ocotillol type; **e** other promiscuous subtype saponins core; **f** other promiscuous subtype saponins core; **g** 24-hydroxy-20(S)protopanaxadiol saponins core; **h** other promiscuous subtype saponins core

**Fig. 3:**
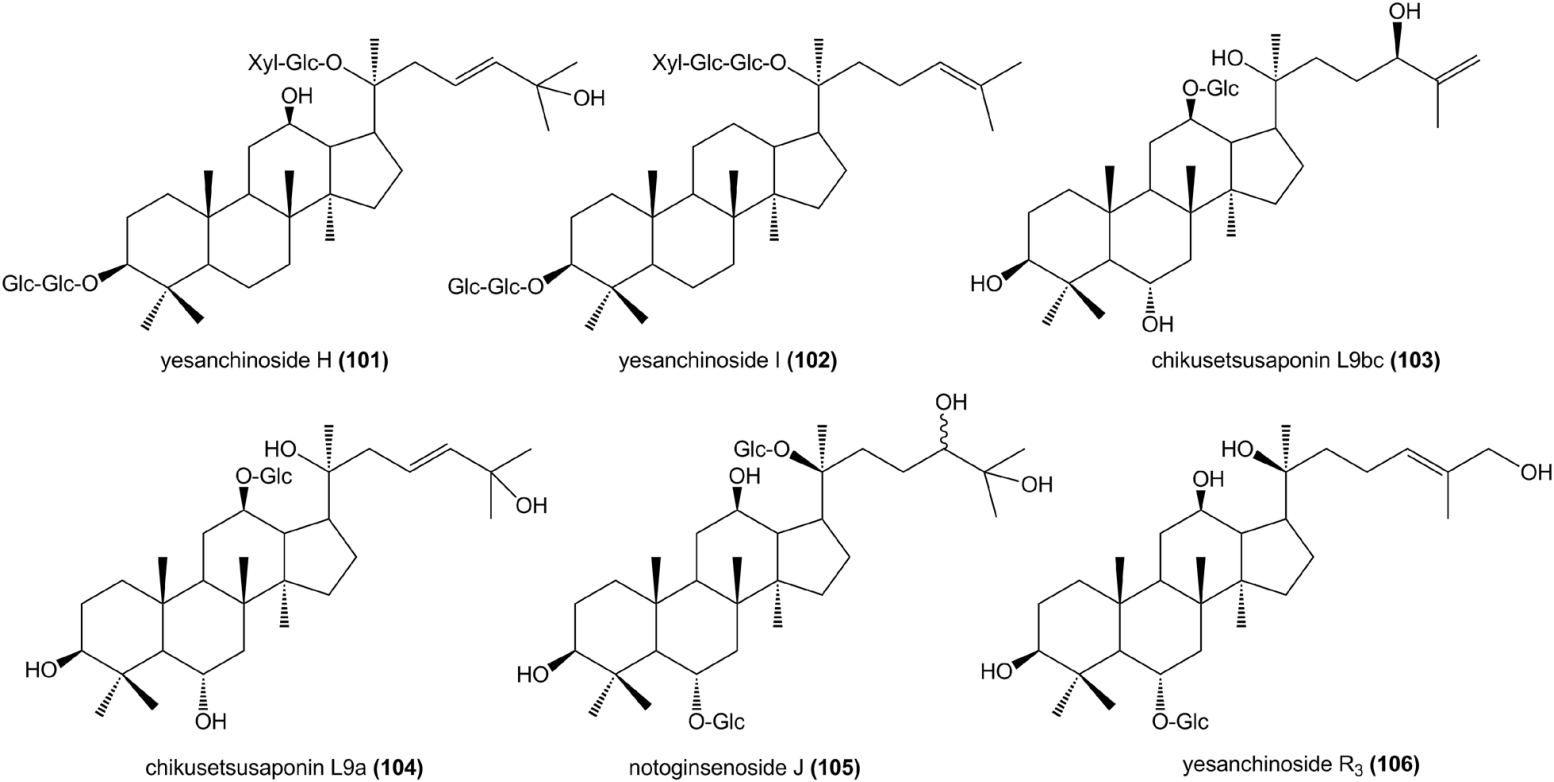
The chemical structures of compounds 101–106 from Zhujieshen

Zhujieshen is a rich source of many bioactive phytochemicals that have been isolated and identified from rhizomes, taproots, lateral roots, leaves, and fruits [7–10]. Triterpenoid saponins are the main bioactive components of Zhujieshen, including oleanolane type (A) and dammarane-type saponins. The dammarane type saponins can be divided into 20(S)-protopanaxadiol type (B) and 20(S)-protopanaxatriol type (C) triterpenoid saponins according to whether the C6 position of the dammarane nucleus contains a hydroxyl group or an O-glycoside substituent [11]. Additionally, there is ocotillol type (D) saponins in Zhujieshen, in which a furan ring is introduced by an oxygen atom connection between C20 and C24 of the dammarane skeleton. These are also known as 20(S), 24(S)-epoxydammarane-3β,6α,12β,25-tetraol type tetracyclic triterpenoid saponins [11]. The parent nuclei of different saponins are shown in Fig. 2. The main saponins in Zhujieshen are shown in Table 1 and Fig. 3. Moreover, other interesting compounds, such as volatile oils and polysaccharides have been reported to contribute to the biological activities of Zhujieshen [1, 7, 25–27]. Analysis of plant volatiles is a significant and growing area of research, in which gas chromatography combined with mass spectrometry has been used to analyze the volatile oils. Table 2 shows the constituents of volatile oils from Zhujieshen [7, 28, 29]. Carbohydrates are important chemical components isolated from Zhujieshen, which include polysaccharides, oligosaccharides, and reducing sugars. Two polysaccharides that activate the reticuloendothelial system, Tachibana-A and B, were isolated from Zhujieshen by Ohtani et al. in 1989 [76]. Huang et al. isolated five water-soluble polysaccharides (RPS1–RPS5) from Zhujieshen. They also determined their average molecular masses and characterized their morphologies. In addition, it is rich in amino acids, and contains a small quantity of inorganic elements and other ingredients [26].

**Table 1:** The saponins isolated from Zhujieshen

No.	Name	Mother nucleus	R1	R2	R3	References
1	Oleanolic acid	A	H	H		[2]
2	Oleanolic acid- 28-O-β-d-glucopyranoside	A	H	Glc		[12]
3	Chikusetsusaponin Ib	A	GlcUA(4 → 1)Ara(6 → 1)Glc	H		[2]
4	Chikusetsusaponin II	A	GlcUA(2 → 1)Glc	H		[13]
5	Chikusetsusaponin IV	A	GlcUA(4 → 1)Ara	Glc		[2]
6	Chikusetsusaponin IV a	A	GlcUA	Glc		[2]
7	Chikusetsusaponin V	A	GlcUA(2 → 1)Glc	Glc		[2]
8	Cynarasaponin C	A	GlcUA	Glc		[14]
9	desglucosylchikusetsusaponin IV	A	GlcUA(4 → 1)Ara(f)	H		[12]
10	Gardeniside C	A	(6-O-methyl)GlcUA	Glc		[15]
11	Ginsenoside Ro	A	GlcUA(2 → 1)Glc	Glc		[16]
12	Hemsgiganoside B	A	GlcUA	Glc(6 → 1)Glc		[15]
13	Polysciassaponin P5	A	GlcUA(2 → 1)Glc	H		[12]
14	Pseudo-ginsenoside-RT_1_	A	GlcUA(2 → 1)Xyl	Glc		[17]
15	Pseudo-ginsenoside-RP_1_	A	GlcUA(2 → 1)Xyl	Glc		[18]
16	pjs-1	A	H	Glc		[11, 19]
17	pjs-2	A	Glc(2 → 1)Xyl	Glc		[11]
18	pjs-4	A	Ara	Glc		[20]
19	28-Desglucosyl-chikusetsusaponin IVa	A	GlcUA	H		[10]
20	Zingibroside R1	A	GlcUA(2 → 1)Glc	H		[9]
21	Betulafolenetriol	B	H	H	H	[15]
22	Chikusetsusaponin Ia	B	Glc(6 → 1)Xyl	H	H	[2]
23	Chikusetsusaponin III	B	Glc(2 → 1)Glc(6 → 1)Xyl	H	H	[2]
24	Chikusetsusaponin VII	B	Glc(6 → 1)Xyl	Glc(6 → 1)Glc	H	[9]
25	Chikusetsusaponin FK_4_	B	Glc(2 → 1)Glc(6 → 1)Xyl	Glc(6 → 1)Ara(f)	H	[10]
26	Chikusetsusaponin FK_5_	B	Glc(2 → 1)Glc(6 → 1)Xyl	Glc(6 → 1)Xyl	H	[10]
27	Chikusetsusaponin FK_6_	B	Glc(2 → 1)Glc(6 → 1)Xyl	Glc	H	[21]
28	Chikusetsusaponin FK_7_	B	Glc(2 → 1)Glc	H	Glc	[8]
29	Chikusetsusaponin VI	B	Glc(2 → 1)Glc(6 → 1)Xyl	Glc(6 → 1)Glc	H	[13]
30	Ginsenoside Rb_1_	B	Glc(2 → 1)Glc	Glc(6 → 1)Glc	H	[16, 22]
31	Ginsenoside Rb_2_	B	Glc(2 → 1)Glc	Glc(6 → 1)Ara(p)	H	[16, 22]
32	Ginsenoside Rb_3_	B	Glc(2 → 1)Glc	Glc(6 → 1)Xyl	H	[8]
33	Ginsenoside Rc	B	Glc(2 → 1)Glc	Glc(6 → 1)Ara(f)	H	[16, 22]
34	Ginsenoside Rd	B	Glc(2 → 1)Glc	Glc	H	[22]
35	Ginsenoside Rg_3_	B	Glc(2 → 1)Glc	H	H	[16]
36	Ginsenoside F_2_	B	Glc	Glc	H	[21]
37	Gypenoside XVII	B	Glc	Glc(2 → 1)Glc	H	[2]
38	Notoginsenoside R_4_	B	Glc(2 → 1)Glc	Glc(6 → 1)Glc(6 → 1)Xyl	H	[13]
39	Notoginsenoside Fa	B	Glc(2 → 1)Glc(2 → 1)Xyl	Glc(6 → 1)Glc	H	[13]
40	Notoginsenoside Fc	B	Glc(2 → 1)Glc(2 → 1)Xyl	Glc(6 → 1)Glc	H	[23]
41	Notoginsenoside Fe	B	Glc	Glc(6 → 1)Ara(f)	H	[21]
42	20(S)-protopanaxadiol	B	H	H	H	[12]
43	Yesanchinoside J	B	(6-O-acetyl)Glc(2 → 1)Glc	Glc(6 → 1)Glc(6 → 1)Xyl	H	[13]
44	6’’’-O-acetyl-ginsenoside Re	C	H	(6-O-acetyl)Glc(2 → 1)Rha	Glc	[12]
45	20(S)-protopanaxatriol	C	H	H	H	[15]
46	20-O-Glu-ginsenoside R_f_	C	H	Glc(2 → 1)Glc	Glc	[23]
47	Chikusetsusaponin LM_1_	C	H	H	Glc(6 → 1)Xyl	[8]
48	Chikusetsusaponin LM_2_	C	H	H	Glc(6 → 1)Xyl(3 → 1)Xyl	[8]
49	Chikusetsusaponin LM_3_	C	H	H	Glc(6 → 1)Ara(3 → 1)Xyl	[8]
50	Chikusetsusaponin LM_5_	C	Glc(2 → 1)Glc	H	Glc(6 → 1)Ara(f)	[8]
51	Chikusetsusaponin LM_6_	C	Glc(2 → 1)Glc	H	Glc(6 → 1)Ara(p)(4 → 1)Xyl	[8]
52	Chikusetsusaponin FK_1_	C	H	Glc(2 → 1)Rha	H	[15]
53	Chikusetsusaponin L5	C	H	H	Glc(6 → 1)Ara(p)(4 → 1)Xyl	[8]
54	Floralquinquenoside E	C	H	Glc(2 → 1)Rha	Glc(6 → 1)Xyl	[15]
55	Ginsenoside Rg_1_	C	H	Glc	Glc	[2, 14]
56	Ginsenoside Rg_2_	C	H	Glc(2 → 1)Rha	H	[2]
57	Ginsenoside Re	C	H	Glc(2 → 1)Rha	Glc	[2]
58	Ginsenoside Rh_1_	C	H	Glc	H	[9]
59	Ginsenoside Rf	C	H	Glc(2 → 1)Glc	H	[22]
60	Ginsenoside F_1_	C	H	H	Glc	[14]
61	Ginsenoside F_3_	C	H	H	Glc(6 → 1)Ara(p)	[8]
62	Ginsenoside F_5_	C	H	H	Glc(6 → 1)Ara(f)	[8]
63	Ginsenoside F_6_	C	Glc	Glc	Glc(6 → 1)Ara(f)	[8]
64	Notoginsenoside R_1_	C	H	Glc(2 → 1)Xyl	Glc	[16]
65	Notoginsenoside R_2_	C	H	Glc(2 → 1)Xyl	H	[2]
66	Notoginsenoside R6	C	H	Glc	Glc(6 → 1)α-Glc	[14]
67	Pseudo-ginsenoside RS1	C	H	(6-O-acetyl)Glc(2 → 1)Rha	Glc	[15]
68	Yesanchinoside D	C	H	(6-O-acetyl)Glc	Glc	[14]
69	Yesanchinoside E	C	H	Glc(2 → 1)Rha	Glc(6 → 1)Glc	[15]
70	Yesanchinoside F	C	H	(6-O-acetyl)Glc(2 → 1)Rha	Glc(6 → 1)Glc	[15]
71	Yesanchinoside A	D	(6-O-acetyl)Glc(2 → 1)Glc			[15]
72	Yesanchinoside B	D	Glc(2 → 1)Glc(6 → 1)α-Glc			[14]
73	Yesanchinoside C	D	Glc(2 → 1)Glc(6 → 1)Xyl			[15]
74	Vina-ginsenoside R_1_	D	(6-O-acetyl)Glc(2 → 1)Rha			[13]
75	Vina-ginsenoside R_2_	D	(6-O-acetyl)Glc(2 → 1)Xyl			[13]
76	Vina-ginsenoside R_6_	D	Glc(2 → 1)Xyl(6 → 1)α-Glc			[23]
77	(24S)-pseudo-ginsenoside F11(24R)	D	Glc(2 → 1)Rha			[17]
78	(24S)-pseudo-ginsenoside RT4	D	Glc			[18]
79	Majonoside R_2_(24S)	D	Glc(2 → 1)Xyl			[13]
80	3-O-acetyl-12-ketoderivative	E	Ac	H	H	[15]
81	Chikusetsusaponin LT_5_	E	Glc	H	Glc(6 → 1)Glc	[15]
82	Chikusetsusaponin LT_8_	E	Glc	H	Glc	[15]
83	Chikusetsusaponin LN_4_	E	Glc(6 → 1)Xyl	H	Glc(6 → 1)Ara(p)	[10, 15]
84	Chikusetsusaponin FK_2_	E	Glc(2 → 1)Glc	H	Glc	[10]
85	Chikusetsusaponin FK_3_	E	Glc(2 → 1)Glc(6 → 1)Xyl	H	Glc	[10]
86	Chikusetsusaponin FH_1_	E	H	OH	Glc(6 → 1)Ara(f)	[10]
87	Chikusetsusaponin FH_2_	E	Glc(2 → 1)Glc(6 → 1)Xyl	H	Glc(6 → 1)Ara(f)	[10]
88	Chikusetsusaponin FT_1_	E	H	OH	Glc(6 → 1)Ara(p)	[10]
89	Chikusetsusaponin FT_2_	E	Glc(2 → 1)Glc(6 → 1)Xyl	H	Glc(6 → 1)Glc	[10]
90	Chikusetsusaponin FT_3_	E	Glc(2 → 1)Glc(6 → 1)Xyl	H	Glc(6 → 1)Ara(p)	[10]
91	Chikusetsusaponin FT_4_	E	Glc(6 → 1)Xyl	H	Glc	[10]
92	Ginsenoside Rh_8_	E	H	OH	Glc	[15]
93	Prosapogenin	E	H	H	Glc	[15]
94	Chikusetsusaponin LM_4_	F	Glc(2 → 1)Glc	H	Glc	[8]
95	Chikusetsusaponin L10	F	H	H	Glc	[8]
96	Yesanchinosides G	G	Glc(2 → 1)Glc	Glc(6 → 1)Xyl		[24]
97	Notoginsenoside G	G	Glc(2 → 1)Glc	Glc		[24]
98	Quinquenoside IV	G	Glc(2 → 1)Glc	Glc(6 → 1)Glc		[24]
99	Yesanchinoside R1	H	Glc			[12]
100	Yesanchinoside R2	H	Glc(2 → 1)Xyl			[12]

**Table 2:**
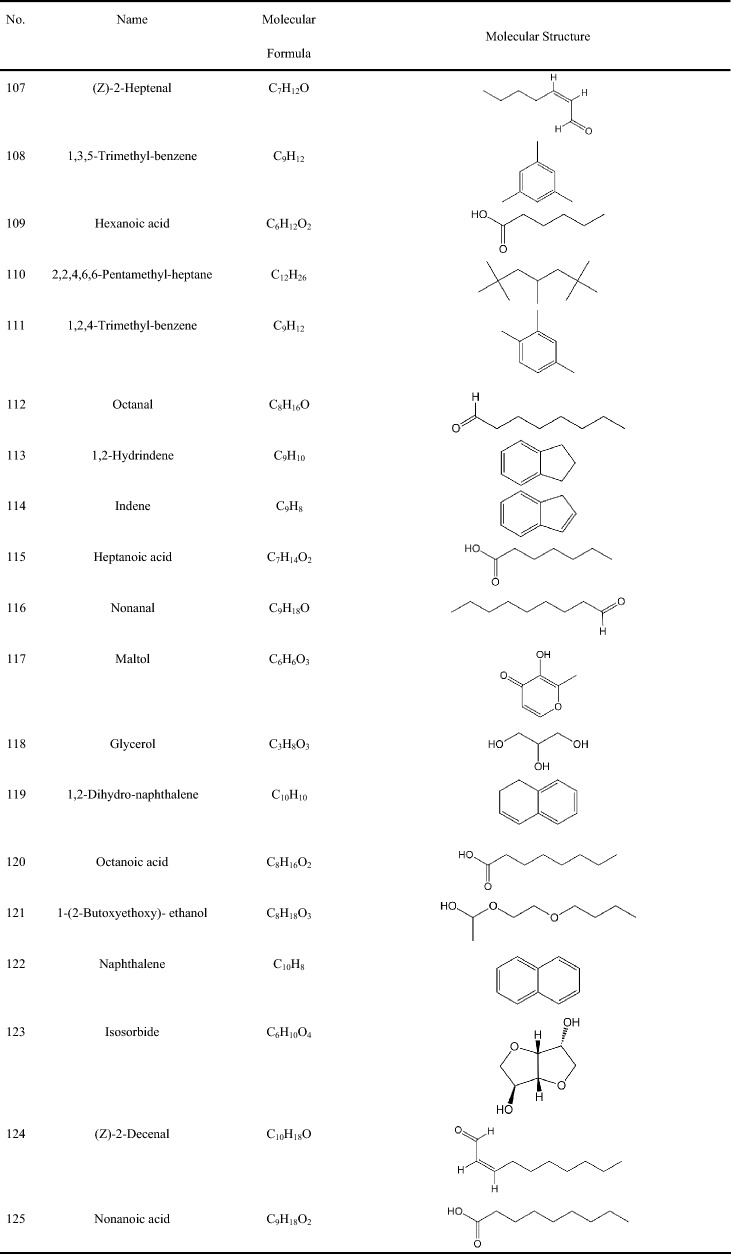

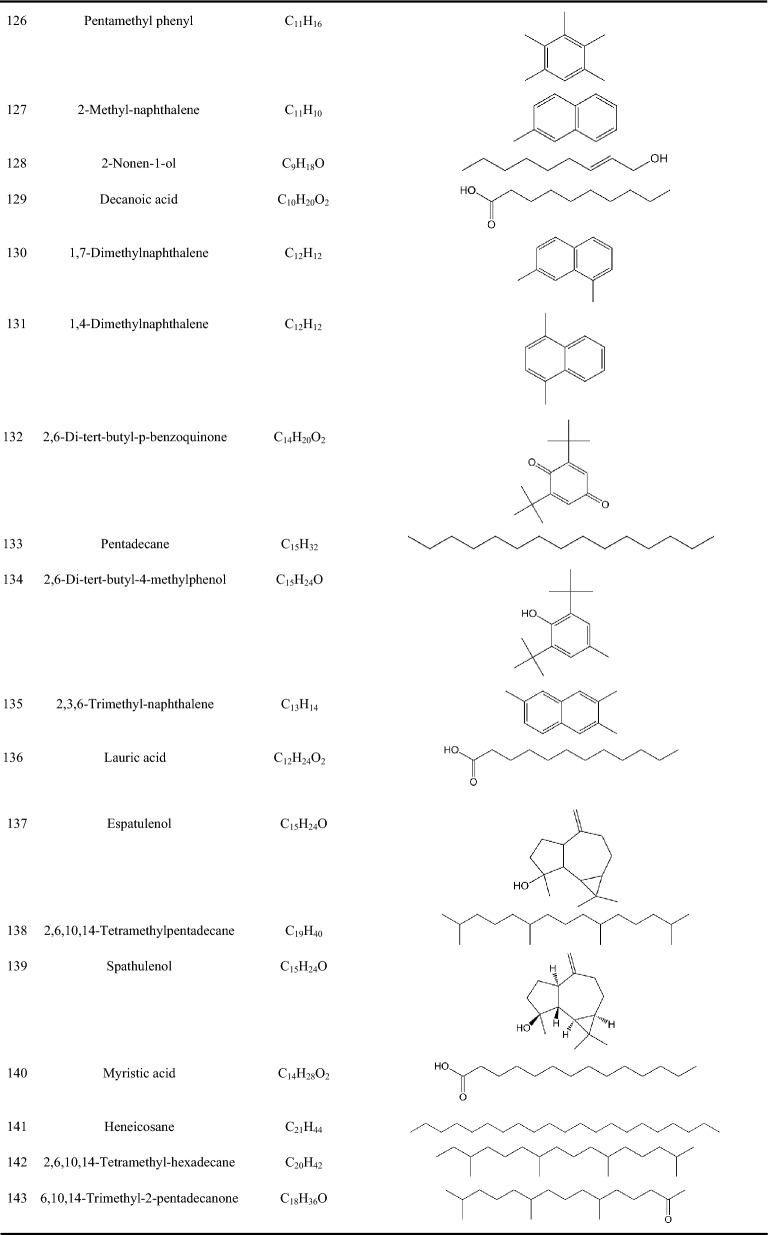

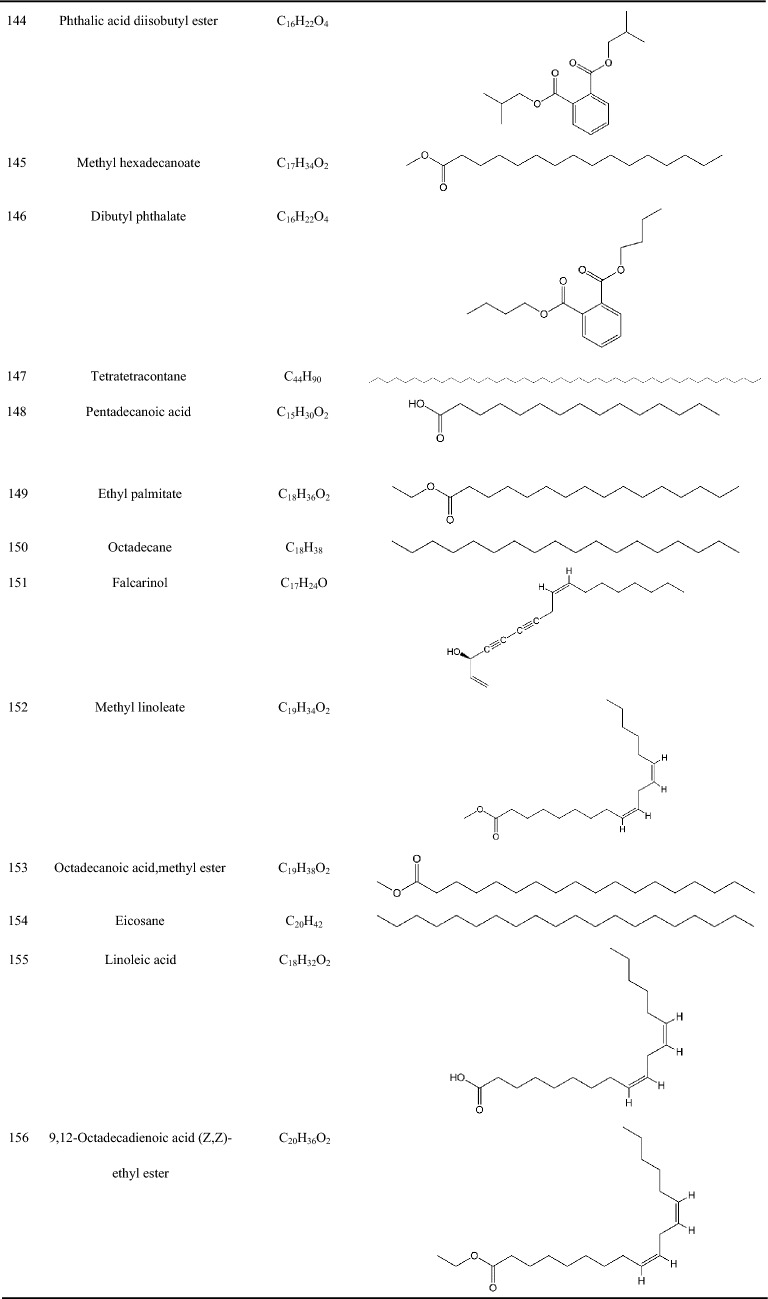

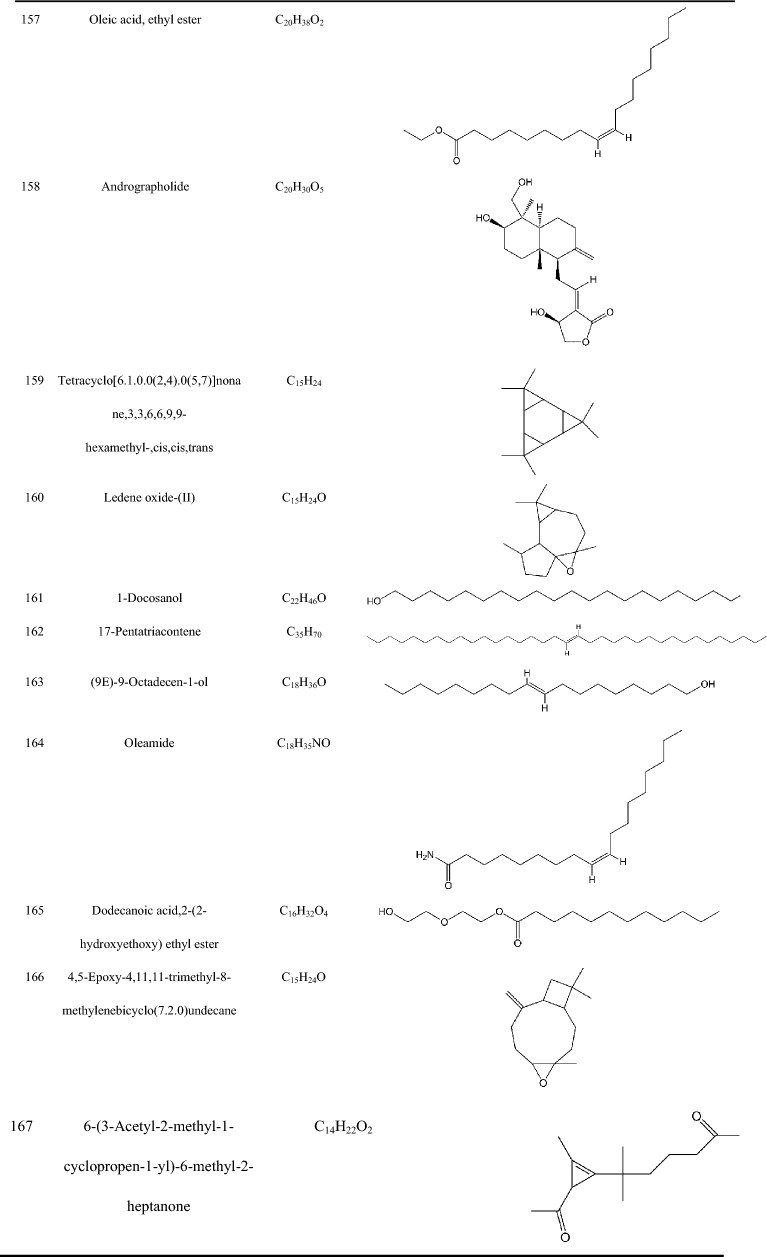

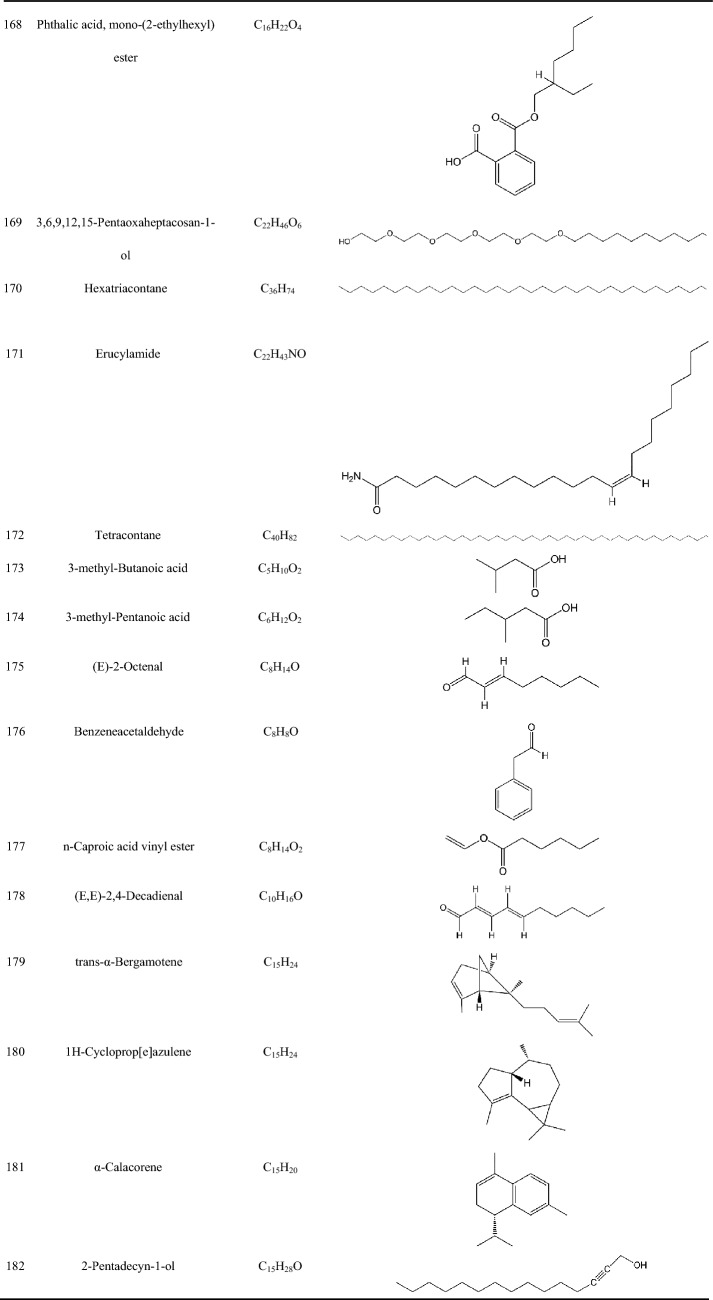
Constituents of the volatile oils from Zhujieshen

## Pharmacological activities

### Anti-inflammatory activity

Increasing lines of evidence indicate the potential of Zhujieshen for effective treatment of inflammatory diseases [30–33]. Saponins from Zhujieshen extract have been found to regulate MAPK and NF-κB signaling pathways to attenuate age-related neuroinflammation [34]. In another study, saponins isolated from Zhujieshen were shown to inhibit inflammation in the colon of natural aging rats, possibly by modulation of the neuraminidase 3/intestinal alkaline phosphatase signaling pathway [35]. Chikusetsusaponin V exhibited anti-inflammatory activity in LPS-stimulated RAW 264.7 macrophages via inhibition of NF-κB and MAPK signaling pathways [36]. Chikusetsusaponin IVa effectively inhibited high-fat diet-induced inflammation in mouse adipose tissue by inhibiting NLRP3 inflammasome activation and NF-κB signaling [37]. Additionally, polysaccharides from Zhujieshen reduced LPS-induced microglia inflammation, possibly by inhibition of NF-κB signaling [38].

### Hepato-protective activity

With the deeper studies of Zhujieshen, it is gradually accepted that it also has hepato-protective activity [39–42]. The hepato-protective activity of total saponins from Zhujieshen was investigated in a mouse model of fatty liver fibrosis, in which the mice were fed a high-fat diet combined with an intraperitoneal injection of porcine serum [43]. The low-dose (100 mg/kg) and high-dose (300 mg/kg) groups of mice on a high-fat diet were treated by oral gavage once every 2 days for 11 weeks. The mice were given porcine serum by intraperitoneal injection from the fifth week. Compared with animals in the model group, the final body weight, liver weight, and liver index were markedly decreased by treatment with total saponins, especially in the high-dose group (*P* < 0.05). Liver steatosis, inflammatory cell infiltration, and collagen fiber generation were significantly improved, which might be due to inhibition of CHOP, endoplasmic reticulum stress response, and JNK-mediated apoptosis and inflammation. Another study found that saponin from Zhujieshen, chikusetsusaponin V, attenuated LPS-induced acute liver injury in mice as a consequence of its potent anti-inflammatory activity [44]. The polysaccharides isolated from Zhujieshen had a protective effect on liver injury induced by acetaminophen and relieved acute liver injury induced by LPS and D-galactosamine. The mechanism for this protective effect may be related to its anti-inflammatory and antioxidant activities [45, 46]. Additionally, studies have indicated that extracts of Zhujieshen could improve non-alcoholic fatty liver induced in mice by high-fat diet [47] and have a preventive effect on alcoholic liver injury [48]. These studies demonstrate that Zhujieshen has potential in the prevention and treatment of liver diseases.

### Cardio-protective effects

Different fractions extracted from Zhujieshen have exhibited highly effective cardiac protection [49]. The cardioprotective effects of saponins from Zhujieshen were evaluated in a rat model of acute myocardial ischemia injury via ligation of the left anterior descending branch [50]. Multiple indicators were utilized to evaluate cardiac protection, including infarct size, biochemical indicators, hemodynamics, influences on myocardial pathology, and mRNA expression of superoxide dismutase, caspase-3, and Bcl-2 families. The results demonstrated that saponins from Zhujieshen exerted beneficial cardiac protection by scavenging oxidative stress-triggered accumulation and overproduction of reactive oxygen species, alleviating myocardial ischemia injury and decreasing cardiac cell apoptosis. The mechanism involved the inhibition of NF-kB, ERK1/2, and p38 MAPK activation, increasing the expression of sirtuin1 to alleviate myocardial infarction injury and cardiac cell apoptosis [51]. Furthermore, chikusetsusaponin IVa, one of the main saponins from Zhujieshen, attenuated myocardial fibrosis induced by isoprenaline, mainly by activating autophagy through the AMPK/mTOR/ULK1 pathway [52]. These results suggest that Zhujieshen could be considered a potential candidate for the treatment of cardiac diseases.

### Neuro-protective effects

The saponins from Zhujieshen conferred neuro-protection in natural aging rats and Alzheimer’s disease rats [53–55]. The mechanism may be partly through the regulation of oxidative stress and mitochondria-related pathways [56]. Saponins, the major components of Zhujieshen, have been reported to exhibit neuroprotective effects against D-galactose-induced neuronal injury by decreasing apoptosis and oxidative stress, ultimately improving cognitive performance [57]. The research revealed that this neuroprotective activity is closely correlated with Nrf2 and SIRT1-mediated anti-oxidant signaling pathways. Chikusetsusaponin V, the most abundant saponin from Zhujieshen, exhibited neuroprotective effects, possibly by modulation of SIRT1/PGC-1α/Mn-SOD signaling pathways [58]. In another study, chikusetsusaponin IVa attenuated isoflurane-induced neurotoxicity and cognitive deficits through SIRT1/ERK1/2 in developmental rats [59]. These findings suggested that Zhujieshen may have great promise for the treatment of neurodegenerative diseases.

### Anti-tumor activity

Cancer is one of the biggest causes of morbidity and mortality in the world, making it an important target for natural medicines with less toxicity [60]. Zhujieshen is well-known for its potential anti-cancer effects. Studies have shown that chikusetsusaponins IVa can also inhibit prostate cancer cell proliferation and induce cell death without cytotoxicity in prostate normal cells. Its mechanism may be through the promotion of intracellular reactive oxygen species (ROS) production, thereby inducing mitochondrial-regulated apoptosis [61]. Furthermore, another study has indicated chikusetsusaponins IV and V exhibit anti-hepatoma effects by influencing apoptosis-related proteins, intracellular calcium levels, and cell proliferation analyzed through CCK-8 [62]. Moreover, Yuan D et al. further verified the inhibitory effect of total saponins of *Panax japonicus* on the growth of HL-60 cells in vitro by culturing HL-60 cells in vitro and detecting the viability and number of cancer cells [63]. In addition to that, the human gastric cancer cell line SGC-7901 [64], murine colon adenocarcinoma CT26 cells [65], mouse H22 hepatoma cells [66, 67], human ovarian cancer A2780 cells [68], human A549 lung cancer cells [69, 70], and human cervical cancer HeLa cells [71, 72] have been used to investigate the anti-tumor activity of Zhujieshen. All of these studies gave positive results that could be attributed to chikusetsusaponins IV, IVa, and V, total saponins, polysaccharides, and their derivatives. The most likely mechanisms include triggering of apoptosis, suppression of migration and invasion of cancer cells, and regulation of oncogene expression [68]. Therefore, Zhujieshen is proposed as a potential adjuvant therapy for protecting against human tumors in the future.

### Antioxidant activity

The extracts obtained from the root of Zhujieshen have exhibited very promising antioxidant activities. The total extracts could eliminate excessive free radicals produced by the body to improve antioxidant capacity and exert anti-apoptotic effects through the Bcl-2 family protein, thereby delaying aging [73]. Similarly, polysaccharides from Zhujieshen showed good potential antioxidant activities by analysis of scavenging capacity for DPPH, hydrogen peroxide, and free radicals of superoxide anion in vitro [74]. Additionally, the essential oil extracted from Zhujieshen has also demonstrated antioxidant activity [75]. These findings suggest that Zhujieshen could be used as a potential treatment for oxidative stress-dependent disorders such as Alzheimer's disease, diabetes mellitus, and arteriosclerosis. However, further research should be conducted in vivo to fully understand its potential and the underlying mechanisms.

### Effect on the immune system

In 1989, it was reported that two polysaccharides from Zhujieshen (Tachibana-A and Tachibana-B) exhibited reticuloendothelial-potentiating activity to improve the activity of macrophages in the reticuloendothelial system [76]. Huang et al*.* found that novel water-soluble highly branched heteropolysaccharides isolated from Zhujieshen were potential immunopotentiators, inhibited the proliferation of S-180 tumor cells, and protected essential organs in BALB/c mice [27]. Moreover, polysaccharides from Zhujieshen significantly improved immune function in Kunming mice with low immunity induced by cyclophosphamide [77–79]. Other immunopharmacological studies revealed that saponins from Zhujieshen exhibited potent stimulating effects in immunosuppressed mice via specific and nonspecific immunity [77–80]. Additionally, a composition of saponins and polysaccharides showed higher immunomodulatory effects than those of saponins or polysaccharides alone [81].

### Effect on the hematological system

In ancient times, *Panax japonicus* had a good effect on diseases caused by “qi” stagnation and blood stasis. Modern studies have shown that Zhujieshen bipinnatifidus could be considered a potential substitute for *P. notoginseng* as a hemostatic herbs. It is mainly used for the treatment of trauma and ischemic cardiovascular diseases [82]. Matsuda et al*.* reported that chikusetsusaponin III, IV, and V showed the promotional effect of the fibrinolytic system [83]. Chikusetsusaponin IVa exerts antithrombotic effects, including minor hemorrhagic events. Research showed that chkusetsusaponin IVa could prolong the recalcification time, prothrombin time, activated partial thromboplastin time, and thrombin time of normal human plasma in a dose-dependent manner, thereby exerting a certain anti-thrombotic effect, including mild bleeding events [84]. Zhang and his team further extract and separate the crude extract of zhujieshen into polysaccharides and small molecular compounds. The research found that both extracted substances could accelerate the recovery of the white blood cell, red blood cell, and hemoglobin levels in the blood deficiency model mice. Hematopoietic activity may result from stimulating the secretion of interleukin-3, interleukin-6, erythropoietin, GM colony-stimulating factor (CSF), and M-CSF and by the resistance of spleen cells to apoptosis [85].

### Other pharmacological effects

The saponins and polysaccharides from Zhujieshen exerted anti-hyperlipidemic potential in a mouse model of hyperlipidemia induced by celiac injection of 75% egg-yolk emulsion [86, 87]. Han et al*.* reported that chikusetsusaponins isolated from Zhujieshen had significant anti-obesity activity in mice fed a high-fat diet, which was partly related to delayed intestinal absorption of fat through inhibition of pancreatic lipase activity [88]. It was found that saponins from Zhujieshen significantly improved reproductive dysfunction in male mice fed a high-fat diet, possibly due to the reduction of macrophage infiltration and inhibition of testicular inflammation mediated by the NF-κB pathway [89]. Some pharmacological studies have found that extracts of Zhujieshen exhibited protective effects on gastric and intestinal mucous membranes [90–92]. It could also alleviate the renal injury of diabetic mice by reducing blood lipids, and regulating the biosynthesis of unsaturated fatty acids and purine metabolism [93]. Furthermore, it has been reported that saponins from Zhujieshen protect against cerebral ischemia injury [94, 95].

## Pharmacokinetics

Studying the pharmacokinetic characteristics of Zhujieshen helps to understand its in vivo behavior and mechanism of action. The pharmacokinetic behaviors of six bioactive saponins (ginsenosides Rb1, Rg1, and Re, chikusetsusaponins V and IV, and hemsgiganoside B) from Zhujieshen were investigated by UHPLC-MS/MS [96]. After oral administration of total saponins at a dose of 500 mg/kg, the main non-compartmental parameters of the six analytes were calculated using DAS 3.2.8 software, including T_1/2_, C_max_, T_max_, AUC_0−t_, CL_z/F_, and apparent volume of distribution. The six analytes were quickly absorbed into the blood, and C_max_ was reached within 0.73 h, except for ginsenoside Rb1 with a C_max_ of 7.60 h. The values of C_max_, T_1/2_, and AUC_0−t_ of ginsenoside Rb1 were significantly greater than those of the others, mainly due to a considerably lower value of CL_z/F_. When saponins extracted from Zhujieshen were orally administered at a dose of 173 mg/kg (equivalent to chikusetsusaponin V 100.3 mg/kg and chikusetsusaponinIV 36.7 mg/kg), similar values of T_1/2_ and T_max_ were obtained for chikusetsusaponin V and chikusetsusaponin IV [97].

## Authentication

Zhujieshen, one of the *Panax* species, is an expensive herbal medicine that is used in China, Korea, Japan, and Vietnam. As a consequence of its high pharmacological and economic value, many plants, including *Phedimus aizoon* (L.)’t Hart (景天三七), *Panax stipuleanatus* H. T. Tsai & K. M. Feng (屏边三七), *Panax japonicus* C. A. Mey. Var. *angustifolius* (Burk) Cheng et Chu (狭叶竹节参) and *Panax japonicus* C. A. Mey. Var. *bipinnatifada* (Seem.) C. Y. Wu et K. M. Feng (羽叶三七), with similar root morphology, have been used to adulterate Zhujieshen [98, 99]. Morphological and histological characteristics are the traditional methods for authentication of herbal medicines, which could be used to authenticate Zhujieshen and its common adulterant (*Phedimus aizoon* (L.)’t Hart.) [100]. However, these methods may not always be precise enough, especially when dealing with closely related species with similar appearances or intra-species morphological variations. Additionally, most commercial ginseng products are solid forms, including powder, granule, pill, capsule, or other processed products, which makes identification difficult. Following advances in the understanding of gene function, DNA barcoding, a biological technique, has been widely used in species identification, breeding, and evolutionary studies [101, 102]. The identification of species-specific DNA markers has played an important role in the authentication of Zhujieshen and related species or adulterants [99, 103, 104]. ITS2 has been successfully established to differentiate Zhujieshen and its non-identical adulterants with a high-rate identification [99]. Choi et al. determined a species-specific amplified fragment length polymorphism-derived sequence for rapid authentication of Zhujieshen among other related *Panax* species [105]. Nguyen et al. developed 18 coding DNA sequence-derived, species-specific, single nucleotide polymorphism markers from chloroplast genomes to authenticate seven *Panax* species (*Panax japonicus*, *Panax ginseng*, *Panax notoginseng*, *Panax stipuleanatus*, *Panax vietnamensis*, *Panax quinquefolius*, and *Panax trifolius*), enabling differentiation of these seven species from the others [106].

## Predictive analysis on quality marker

The combination of microscopic and physicochemical identification is an important tool for identification when Zhujieshen is processed into different products in the Chinese Pharmacopoeia Commission (2020). Given that ginsenoside Ro and chikusetsusaponin IVa are the characteristic ingredients of Zhujieshen, many investigations have used them as biomarkers to determine its quality. According to the reports, triterpenoid saponins compounds including chikusetsusaponin V, chikusetsusaponin IVa, and chikusetsusaponin IV, have multiple activities associated with the efficacy of Zhujieshen, including anti-tumor, anti-inflammatory, and anti-myocardial ischemia effects. Moreover, these compounds have stable activity, can be quantitatively measured, and possess traceability properties [107, 108]. According to the concept of quality markers (Quality markers) proposed by Academician Liu Changxiao, triterpenoids can be the Q-marker of Zhujieshen [109].

## Conclusion and future prospects

**Fig. 4:**
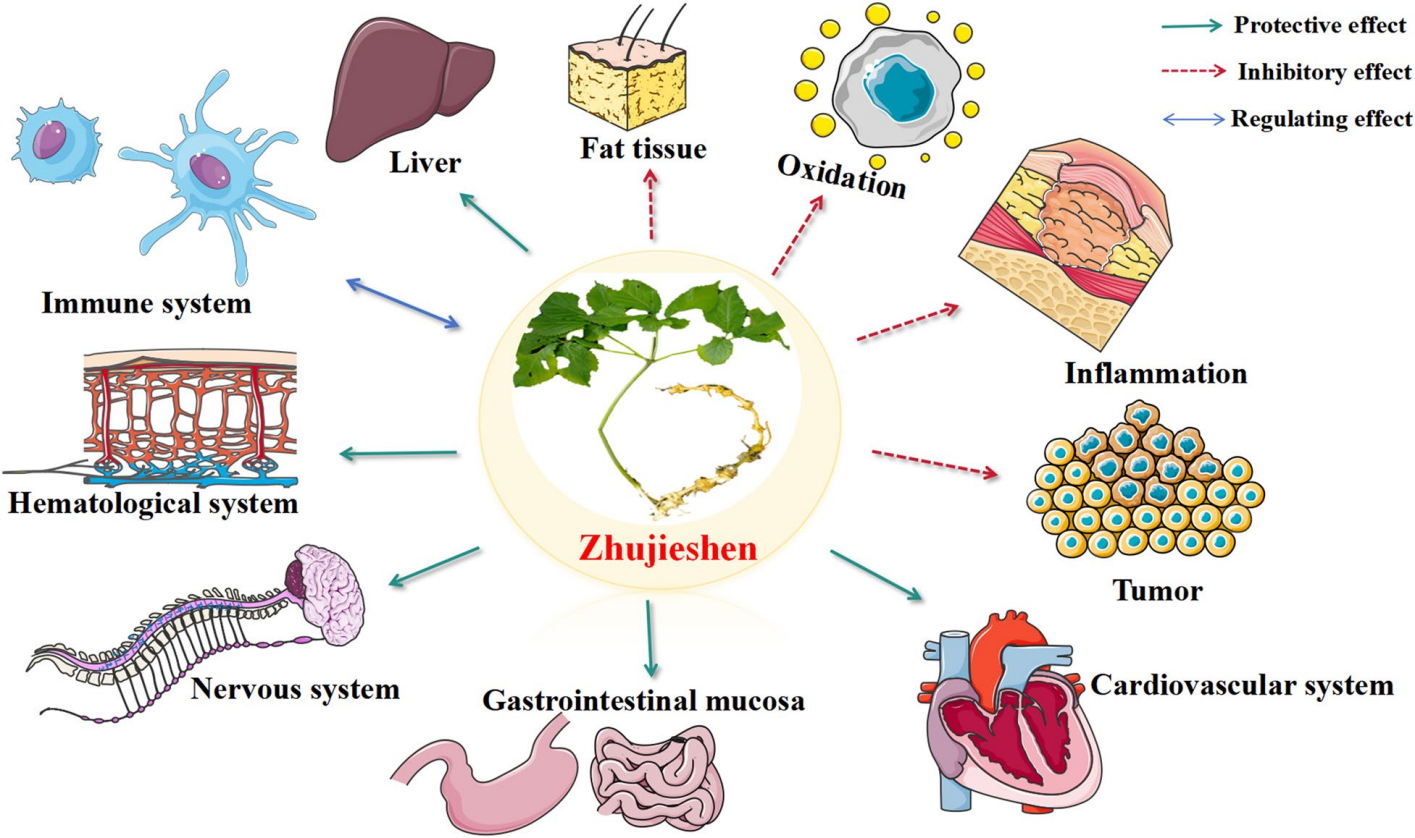
Pharmacological properties of Zhujieshen

It is reported that Zhujieshen possesses both the conserving vitality activities of *Panax ginseng* C. A. Mey and the replenishing blood activities of *Panax notoginseng* (Burkill) F. H. Chen ex C. H. simultaneously [110]. It was traditionally used as *Panax ginseng* to enhance immunity. Additionally, it can be used in the treatment of rheumatic arthritis as *Panax notoginseng*. This is why Zhujieshen was also referred to as the "king of herbs" in traditional Tujia and Hmong medicines. Also, it has been successfully used in clinical practice for centuries to treat fractures, hematemesis, cough, bleeding wounds, arthralgia, and weakness after illness. Phytochemical research has indicated that saponins and polysaccharides are the major active constituents. The total saponin content in the roots of Zhujieshen can reach 15%, which is 2 to sevenfold higher than that of *P. ginseng*. Various types of saponins have been isolated and a range of pharmacological studies have focused on these components. The crude extracts and pure compounds from Zhujieshen have been shown to have diverse pharmacological activities including anti-inflammatory, hepato-protective, cardio-protective, neuro-protective, anti-tumor, anti-oxidant, anti-thrombotic and immunomodulatory activities (Fig. 4). Recent studies have also focused on the potential of Zhujieshen in oxidative stress-dependent disorders such as Alzheimer’s disease, diabetes mellitus, and arteriosclerosis. Although there are many promising results, most studies have only been conducted in cell lines in vitro or animal models. Mechanism of action studies are very limited and no clinical trials of Zhujieshen extracts have been reported yet. Further research on components and their mechanisms of action is needed to advance the therapeutic role of Zhujieshen. Additionally, pharmacological investigation of new saponins and volatile oils, and the exact structural characteristics of polysaccharides and trace compounds isolated from Zhujieshen have mostly been ignored, limiting the diversity of research and application of Zhujieshen. Furthermore, few modern studies have been conducted to confirm any side effects or toxicity. Finally, future research work needs to be focused on the identification of components, further pharmacological studies, and therapeutic mechanisms, together with links between traditional uses, active compounds, and reported pharmacological activities. Such studies would help confirm clinical efficacy and enable improved quality control based on the active compounds. This review might be helpful to summarize the current status of research on Zhujieshen and point out directions for future research.

## Data Availability

Not applicable.

## Abbreviations

AMPK: Adenosine monophosphate-activated protein kinase
AUC_0−t_: Mean residence time, area under the concentration–time curve
CHOP: C/EBP homologous protein
CL_z/F_: Apparent clearance
C_max_: Maximum plasma concentration
CSF: Colony-stimulating factor
DPPH: 2,2-Diphenyl-1-picryl-hydrazyl
ERK1/2: Extracellular regulated protein kinases 1/2
JNK: P-Jun N-terminal kinase
LPS: Lipopolysaccharide
MAPK: Mitogen-activated protein kinase
Mn-SOD: Manganese superoxide dismutase
mTOR: Mammalian target of rapamycin
NF-κB: Nuclear factor-kappa B
NLRP3: Nod-like receptor protein 3
Nrf2: Nuclear factor erythroid 2-related factor 2
PGC-1α: Peroxisome proliferator-activated receptor gamma-coactivator-1α
Q-marker: Quality marker
ROS: Reactive oxygen species
SIRT1: Silent mating type information regulation 2 homologue
T_1/2_: Elimination half-life
T_max_: Time to reach C_m__a__x_
ULK1: Uncoordinated-51-like kinase1
